# Guidelines for the assessment and control of mutagenic impurities in pharmaceuticals

**DOI:** 10.1186/s41021-025-00349-5

**Published:** 2025-12-23

**Authors:** Masamitsu Honma

**Affiliations:** National Institute of Health Sciences (NIHS), 3-25-26 Tonomachi, Kawasaki, Kanagawa 210-9501 Japan

**Keywords:** Ich m7 guideline, Mutagenic impurities, Ames test, Acceptable intake (AI), Threshold of toxicological concern (TTC), N-nitrosamines

## Abstract

Pharmaceuticals may contain trace impurities unrelated to therapeutic activity, which may occur as synthetic intermediates or degradation products. Among these impurities, mutagenic impurities are of particular concern, as even very low levels of exposure can increase the risk of cancer in humans. To address this issue, the International Council on Harmonization (ICH) developed the M7 guideline in 2014, which aim to provide a scientific and practical framework for the assessment and control of mutagenic impurities in pharmaceuticals. The M7 guideline has been updated twice, in 2017 (R1) and 2023 (R2), with the addition of addendum defining compound-specific acceptable intake levels and a comprehensive Q&A document to promote harmonized implementation. Recent cases involving N-nitrosamine impurities have highlighted the need for improved risk assessment methods due to their high carcinogenic potential. The latest revision of the M7 guidelines (M7(R3)) will introduce a new approach to consider the quantitative carcinogenic potential of N-nitrosamines based on their structural characteristics.

## Background

Pharmaceutical products may contain trace amounts of impurities that are unrelated to the therapeutic effects of the active pharmaceutical ingredient (API). These impurities may originate from intermediates generated during synthesis or from degradation products formed within the formulation. Traditionally, the safety evaluation of such impurities has been regulated by the ICH Q3A (R2) guideline “Impurities in New Drug Substances” and the ICH Q3B (R2) guideline “Impurities in New Drug Products” [1, 2]. These guidelines establish threshold levels for impurities based on the maximum daily dose, above which qualification of safety is required.

However, if an impurity is found to be mutagenic, it is generally assumed that mutagenicity has no threshold. Therefore, even exposure to very low levels could theoretically increase the risk of cancer. Accordingly, the general impurity thresholds defined in ICH Q3A and Q3B cannot be applied to mutagenic impurities. To address this issue, the International Council for Harmonisation of Technical Requirements for Pharmaceuticals for Human Use (ICH) developed the guideline ICH M7 in June 2014, titled “Assessment and Control of DNA Reactive (Mutagenic) Impurities in Pharmaceuticals to Limit Potential Carcinogenic Risk [3].” The M7 guideline provides a practical framework for identifying, categorizing, assessing, and controlling DNA-reactive (mutagenic) impurities to minimize potential carcinogenic risk in humans. As a multidisciplinary guideline (“M”), M7 integrates both safety and quality aspects. This article focuses solely on the safety perspective.

Following its initial release, M7 was revised twice. The first revision, ICH M7(R1) (March 2017), included an addendum defining acceptable intake (AI) levels and calculation methods for 14 mutagenic or carcinogenic compounds frequently detected in pharmaceutical manufacturing [4]. The second revision, ICH M7(R2) (April 2023), added seven more compounds and introduced minor textual updates [5, 6]. Moreover, after several years of implementation of M7, variations in interpretation and application were observed among regulatory authorities. To address these inconsistencies and promote consistent implementation, the ICH published a Questions and Answers (Q&A) document in May 2022 [7].

## Scope of the M7 guideline

The ICH guidelines are primarily intended to provide harmonized principles for the development and marketing authorization of new drug substances and new drug products. Accordingly, the ICH M7 guideline applies mainly to new active substances and formulations during clinical development and at the time of marketing authorization applications. Therefore, M7 does not generally apply to already marketed products, except in cases where changes in the synthesis route, formulation, composition, or manufacturing process may affect carcinogenic risk, or when modifications are made to indications or routes of administration.

Furthermore, the guideline is not applicable to APIs and formulations intended for the treatment of “advanced cancer” as defined in the scope of ICH S9 [8]. Similarly, it does not apply to flavoring agents, colorants, fragrances, or excipients that have been previously used in approved pharmaceutical products. However, if a new excipient is being introduced for the first time in a drug formulation and is chemically synthesized, the impurities contained in that excipient are subject to assessment under the M7 guideline.

## General principles

### Target substances

The ICH M7 guideline applies exclusively to DNA-reactive substances that can directly damage DNA, induce mutations, and potentially cause cancer. These are collectively referred to as mutagenic impurities. Mutagenicity refers to a substance’s ability to induce mutations—a critical mechanism in chemical carcinogenesis—and is typically a result of direct chemical interaction between the substance and DNA. Mutations represent irreversible and permanent changes in the genome, and even a single mutation can potentially lead to the initiation of carcinogenesis. Therefore, mutagenicity is generally regarded as a non-threshold phenomenon.

The term genotoxicity, by contrast, has a broader meaning. It encompasses any adverse effect on the genetic material, including DNA damage or chromosomal alterations, that may or may not result in mutation. Thus, a compound that is genotoxic but not mutagenic may cause DNA damage, but not necessarily lead to heritable mutations or cancer. Non-mutagenic genotoxic effects are often considered to have definable thresholds. Earlier regulatory documents, such as those issued by the EMA-CHMP (2006) [9], the US-FDA (2008) [10], and PhRMA [11], used the terms genotoxic and mutagenic interchangeably. However, ICH M7 clarified this distinction by defining its scope strictly as “mutagenic impurities.” In this article, the term mutagenicity is also used consistently.

Mutagenic carcinogens with such mechanisms are generally detected using the bacterial reverse mutation test (Ames test) [12, 13]. In contrast, other types of genotoxic substances that are not mutagenic usually possess threshold effects and do not pose a carcinogenic risk at impurity levels typically encountered in pharmaceuticals. For example, compounds that test positive in chromosomal aberration or micronucleus assays but are negative in the Ames test are outside the scope of ICH M7. Therefore, the Ames test serves as the essential assay for evaluating the mutagenic potential of pharmaceutical impurities to support risk assessment for human carcinogenicity. Alternatively, if the Ames test data are unavailable, structure-based evaluation may be performed using well-established knowledge and predictive tools. Such assessments include literature reviews, category approaches, and computer-based quantitative structure–activity relationship (QSAR) predictions.

### Risk assessment and control of mutagenic impurities

Generally, the carcinogenicity of chemical substances is evaluated through rodent carcinogenicity tests. For mutagenic substances for which no carcinogenicity test data exists, however, the concept of a threshold of toxicological concern (TTC) has been introduced to define an AI. Even if an impurity is a mutagenic carcinogen, if human exposure is below the TTC, the cancer risk is considered negligible. ICH M7 defines the TTC value as 1.5 μg/day. The TTC concept originated from the FDA as a “threshold of regulation” for assessing exposure to genotoxic carcinogens that might migrate from food-contact materials such as plastics or packaging [14]. The TTC value was derived from an extensive analysis of carcinogenicity data on over 1000 chemicals [15]. The analysis estimated that, assuming approximately 10% of all chemicals are carcinogenic, for 99% of the chemicals, a daily exposure of 1.5 μg/day would ensure a lifetime excess cancer risk of less than 1 in 100,000 (10^− 5^) [16]. However, certain chemical classes are known to be highly carcinogenic and may pose a significant cancer risk even at levels below the TTC. These are collectively known as “Cohorts of Concern (COC)”, including aflatoxin-like compounds, N-nitroso compounds, and alkylazoxy compounds. The general TTC value (1.5 μg/day) is not appropriate for these COC chemicals.

The TTC-based approach defined in ICH M7 thus provides a framework that enables practical management of mutagenic impurities while ensuring patient safety. While carcinogenic risk assessment is generally based on lifetime exposure, in reality, many people may not take the same pharmaceutical continuously throughout their lives. Therefore, for short periods of exposure, a higher AI limit may be justified by considering total exposure.

Because the TTC is a highly conservative approach, even a slight increase in the 1.5 μg/day limit does not necessarily imply a significant increase in cancer risk. Furthermore, if an impurity is shown to be non-carcinogenic in rodent carcinogenicity studies, the substance is excluded from the scope of ICH M7 and there is no concern about carcinogenicity.

## Hazard assessment

### Ames test and follow-up of the positive result

The mutagenic potential of an impurity should, in principle, be assessed using the bacterial reverse mutation test (Ames test). The assay should be conducted in accordance with the ICH S2(R1) guideline and the OECD Test Guideline 471, employing an appropriate and scientifically valid protocol [17, 18]. While the test should ideally comply with Good Laboratory Practice (GLP) standards, certain deviations may be acceptable due to the specific nature of impurities. For instance, GLP compliance may not be feasible when sample preparation or analytical verification cannot be performed under GLP conditions, or when isolation or synthesis of the impurity is technically difficult, preventing testing at the highest concentrations specified in the guideline. In such cases, the use of miniaturized or scaled-down versions of the Ames test (often referred to as “mini-Ames tests”) with a limited number of tester strains has been explored for small sample quantities [19]. Whether such data are acceptable depends on the judgment of the regulatory authority.

A positive result in the Ames test provides critical evidence that the impurity is mutagenic. However, a positive response may sometimes reflect bacterial-specific mechanisms that are not relevant to humans. In such situations, in vivo follow-up assays are required to clarify whether the mutagenic response observed in vitro has biological significance in vivo. The ICH S2(R1) guideline notes that a positive Ames result indicates DNA reactivity, and unless justified by an appropriate risk–benefit analysis, additional in vivo studies are warranted to further assess potential mutagenicity or carcinogenicity [17]. Recommended follow-up tests include transgenic rodent gene mutation assays (TGR) and Pig-a assays, which detect gene mutations in somatic or blood cells, respectively.

The TGR assay, as described in OECD Test Guideline 488, is highly informative for evaluating in vivo mutagenicity in target organs such as the liver or stomach [20]. The Pig-a assay (OECD TG 470) uses peripheral blood cells and is suitable for detecting systemic mutagenic effects [21]. However, because the Pig-a assay is based on peripheral blood sampling, it may not be appropriate for assessing unstable mutagenic metabolites that are formed locally through metabolic activation in the liver.

In recent years, error-corrected next-generation sequencing (ecNGS) has attracted attention as a potential alternative to TGR assays [22, 23]. The ecNGS approach enables comprehensive detection of induced mutations across the entire genome. A major advantage of this method is that DNA samples obtained from existing toxicological studies—such as repeated-dose toxicity or carcinogenicity studies—can be reused for mutation analysis without the need for additional animal testing. This leads to a significant reduction in animal use and represents an important advancement from the perspective of animal welfare. The incorporation of ecNGS-based mutation assays into future OECD Test Guidelines is currently under consideration [24].

### Quantitative structure–activity relationship (QSAR) analysis

The ICH M7 guideline recommends the use of QSAR approaches as an alternative to experimental Ames testing for evaluating the mutagenic potential of pharmaceutical impurities. Although the Ames test prediction is inherently qualitative, the term “QSAR” (or more precisely, “(Q)SAR”) is commonly used to describe structure-based predictive models for mutagenicity. It is noteworthy that ICH M7 represents the first international guideline to formally endorse the application of QSAR as alternatives to biological assays for the purpose of human health risk assessment. In many cases, impurities in pharmaceuticals are present only at trace levels, may consist of multiple chemical types, or are chemically unstable. Consequently, isolating or synthesizing sufficient quantities for biological testing is often difficult or impossible. In such circumstances, once the chemical structure is known, structure-based in silico prediction is a very practical and scientifically sound approach to assess mutagenicity. ICH M7 requires the application of two complementary QSAR prediction methods:

1. Expert rule-based models – which identify structural alerts associated with mutagenicity based on mechanistic or empirical knowledge.
2. Statistical-based models – which use machine learning or regression approaches derived from large curated Ames test datasets.

Currently, more than twenty QSAR tools (both commercial and public) are available worldwide for predicting Ames mutagenicity. Although ICH M7 does not endorse any specific tool, any QSAR model may be used provided that it meets quality criteria and has been developed and validated in accordance with the OECD principles for QSAR validation. A QSAR Model Reporting Format (QMRF) document describing the model’s algorithm, domain of applicability, and validation results must be submitted as supporting information.

If both complementary QSAR systems indicate no structural alerts, the impurity can be concluded to have no mutagenic concern, and no further testing is required. Conversely, if the two systems produce discordant or inconclusive predictions, additional justification, expert review, or experimental follow-up (e.g., Ames test) is necessary to reach a final conclusion. Therefore, the final determination of mutagenic potential may rely on both QSAR outcomes and expert scientific judgment. QSAR under ICH M7 should not be viewed merely as a predictive screening tool for Ames test’s outcomes but as a scientifically equivalent approach to actual experimental mutagenicity testing when applied correctly.

When ICH M7 was first established in 2014, the predictive performance of QSAR tools was still limited, with reported sensitivities below 50% for novel chemical entities [25]. However, following extensive model improvement efforts, including the international Ames/QSAR Challenge Project, which aimed to expand training datasets and harmonize evaluation procedures, the average predictive accuracy of many QSAR tools has improved to approximately 85% [26, 27]. Several tools have since been optimized specifically for compliance with ICH M7 and have been widely implemented by the pharmaceutical industry for impurity assessments [28].

### Classification and control of impurities

In hazard assessment under the ICH M7 framework, available carcinogenicity and mutagenicity data for each impurity (chemical substance) should first be collected through database and literature searches. Based on these data, impurities are classified into one of five categories (Classes 1–5), as follows;

- Class 1: Compounds with established carcinogenicity data allowing derivation of compound-specific AI based on TD₅₀ value (the chronic dose inducing tumors in 50% of animals in carcinogenicity studies).
- Class 2: Mutagenic compounds without adequate carcinogenicity data; these are managed according to the TTC concept (1.5 μg/day).
- Class 3: Compounds for which no data are available from either carcinogenicity or Ames test. For these, QSAR-based Ames mutagenicity predictions must be performed to assess potential mutagenicity. If the QSAR predicts no structural alerts, the impurity may be regarded as non-mutagenic (Class 5) and managed as a regular impurity under ICH Q3A/B. If the QSAR indicates mutagenic potential, the impurity should be managed according to the TTC (Class 2) or tested experimentally using the Ames test to confirm its mutagenicity.
- Class 4: Compounds treated as non-mutagenic; these have quite similar structure of API in which have been tested and were non-mutagenic.
- Class 5: Compounds known to be non-mutagenic based on robust data or established chemical knowledge; these are also excluded from M7 and treated under standard impurity guidelines (ICH Q3A/B).

Through this classification system, ICH M7 provides a framework for the efficient and rational management of mutagenic impurities depending on the range of available scientific information. In particular, the use of QSAR and TTC for Class 3 and Class 2 impurities plays an important role as a scientific risk management approach when experimental data is limited.

## Risk characterization

### Acceptable intake (AI) based on compound-specific risk assessment

**Fig. 1:**
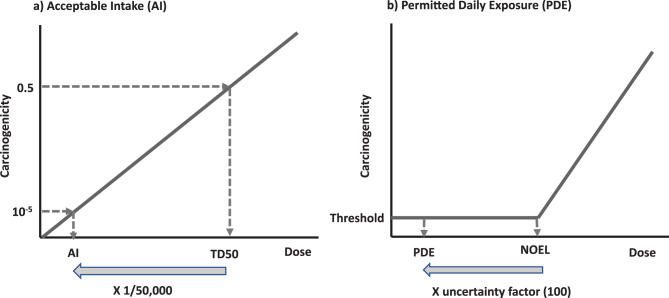
**a** decision tree summarizing the overall framework for assessment and control mutagenic impurities under ICH M7

When rodent carcinogenicity data are available for an impurity, an individual compound-specific AI should be derived rather than applying the general TTC value. The AI can be calculated from the TD₅₀ value—the chronic dose that induces tumors in 50% of the test animals in carcinogenicity studies (Fig. 1a). Alternatively, AI values can be derived using other validated quantitative risk assessment methods used by regulatory authorities, or existing published AIs from regulatory authorities can be used if available.

The compound-specific AI approach allows for more accurate risk characterization ensuring that impurities with known carcinogenic properties are controlled at levels consistent with a lifetime excess cancer risk of 1 × 10^− 5^ (1 in 100,000), in line with the principle of proportional risk management in ICH M7.

### Mitigating risk levels based on structure

For certain classes of mutagenic impurities that share common structural features with well-characterized compounds, it is scientifically acceptable to derive AI limits based on the carcinogenicity data of analogous chemicals. For example, monoalkyl chlorides are a structural class for which multiple compounds have established TD₅₀ values. Analysis of these data indicates that their calculated AIs typically exceed the TTC (1.5 μg/day) by more than 30-fold [29]. Therefore, for this specific structural class, a higher AI of 15 μg/day is considered scientifically justified. This structure-based adjustment recognizes that some chemical classes exhibit consistently low carcinogenic potency, allowing for pragmatic relaxation of limits while maintaining an adequate margin of safety. Such read-across approaches should be scientifically justified, supported by reliable carcinogenicity data for the analogues used, and documented transparently in the impurity risk assessment report.

### Acceptable intake (AI) for less-than-lifetime (LTL) exposure

According to Haber’s rule, a fundamental concept in toxicology, the risk posed by a mutagenic carcinogen is dependent on the total cumulative exposure, where the relationship between concentration (C) and exposure duration (T) is expressed as C × T = constant (k). Based on this principle, AI values can be mitigated for less-than-lifetime (LTL) exposure periods [30]. The default TTC value of 1.5 μg/day represents the daily exposure considered safe over a lifetime of 70 years. Therefore, the total lifetime exposure (1.5 μg/day × 365 days × 70 years = 38.3 mg) can be redistributed proportionally across shorter treatment durations. This approach allows higher daily AI limits for shorter exposures while maintaining an equivalent lifetime cumulative dose. Table 1 shows LTL-based TTC (LTL-TTCs) according to the duration of administration. This approach is applicable not only to marketed products but also to pharmaceuticals during the clinical trial, where the therapeutic benefit has not yet been established. The LTL-TTC approach applies to both continuous and intermittent dosing regimens. For intermittent administration, the total number of dosing days—not the overall treatment duration—should be used to determine the allowable daily intake. For instance, in a regimen of once-weekly dosing for two years (104 total dosing days), the AI corresponds to 20 μg/day. Even for Class 1 compounds, the same proportional adjustment can be applied to derive short-term AI levels consistent with the exposure duration, as shown in Table 1.

**Table 1:** Acceptable intakes for an individual impurity

Duration of treatment	< 1 month	> 1 - 12 months	> 1 - 10 years	> 10 years to lifetime
Daily intake［μg/day］	120	20	10	1.5

In Phase I clinical studies lasting ≤14 days, strict application of LTL-TTC is not required. Only impurities classified as Class 1, Class 2, or belonging to the COC require management according to ICH M7; impurities without structural alerts or those in Class 3 can be treated as non-mutagenic impurities during such limited exposures.

For marketed pharmaceuticals, the LTL-TTC concept may also be applied based on the expected duration of treatment for the majority of patients. Table 2 provides illustrative examples of clinical use scenarios for applying the LTL concept, which may be revised as clinical practices evolve. For example, as a result of major advances in HIV therapy, treatment duration was extended from “1–10 years” to “> 10 years to lifetime” in the ICH M7(R2) revision, reflecting improved patient prognosis and long-term management [5].

1. This table shows general examples; each example should be examined on a case-by-case basis. For example, 10 μg/day may be acceptable in cases where the life expectancy of the patient may be limited e.g., severe Alzheimer’s disease, even though the drug use could exceed 10 years duration
2. Intermittent use over a period > 10 years but based on calculated cumulative dose it falls under the > 1-10 years category
3. HIV is considered a chronic indication but resistance develops to the drugs after 5-10 years and the therapy is changed to other HIV drugs

**Table2:** Examples of clinical use scenarios with different treatment durations for applying acceptable intake (AI)

Duration of treatment	**Examples**^**1**^	AI (μg/day)
≤1 month	Drugs used in emergency procedures (antidotes, anesthesia, acute ischemic stroke), actinic keratosis, treatment of lice	120
> 1 - 12 months	Anti-infective therapy with maximum up to 12 months treatment (HCV), parenteral nutrients, prophylactic flu drugs (~5 months), peptic ulcer, Assisted Reproductive Technology (ART), pre-term labor, preeclampsia, pre-surgical (hysterectomy) treatment, fracture healing (these are acute use but with long half-lives)	20
> 1 - 10 years	Stage of disease with short life expectancy (severe Alzheimer’s), non-genotoxic anticancer treatment being used in a patient population with longer term survival (breast cancer, chronic myelogenous leukemia), drugs specifically labeled for less than 10 years of use, drugs administered intermittently to treat acute recurring symptoms^2^ (chronic Herpes, gout attacks, substance dependence such as smoking cessation), macular degeneration, HIV^3^	10
> 10 years to lifetime	Chronic use indications with high likelihood for lifetime use across broader age range (hypertension, dyslipidemia, asthma, Alzheimer’s (except severe Alzheimer disease), hormone therapy (e.g., growth hormone, thyroid hormone, parathyroid hormone), lipodystrophy, schizophrenia, depression, psoriasis, atopic dermatitis, Chronic Obstructive Pulmonary Disease (COPD), cystic fibrosis, seasonal and perennial allergic rhinitis	1.5

1 This table shows general examples; each example should be examined on a case-by-case basis. For example, 10 μg/day may be acceptable in cases where the life expectancy of the patient may be limited e.g., severe Alzheimer’s disease, even though the drug use could exceed 10 years duration

2 Intermittent use over a period > 10 years but based on calculated cumulative dose it falls under the > 1-10 years category

3 HIV is considered a chronic indication but resistance develops to the drugs after 5-10 years and the therapy is changed to other HIV drugs

### Acceptable intake (AI) for multiple mutagenic impurities

In general, the TTC value applies individually to each mutagenic impurity. When two Class 2 or Class 3 impurities are present in a drug substance or product, the TTC (1.5 μg/day) should be applied separately to each impurity. If three or more mutagenic impurities are present, the sum of their daily intakes must not exceed the total TTC value specified in Table 3. However, in all cases, the AI for each individual impurity must not exceed its corresponding limit derived from the TTC or compound-specific AI. For fixed-dose combination products, impurity control should be applied separately to each active ingredient, as the impurities are specific to the synthesis and formulation of each drug substance. This cumulative approach ensures that the overall mutagenic risk from multiple low-level impurities remains negligible while maintaining the practical feasibility of impurity control in complex drug formulations.

**Table 3:** Acceptable total daily intakes for multiple impurities

Duration of treatment	< 1 month	> 1 - 12 months	> 1 - 10 years	> 10 years to lifetime
Daily intake ［μg/day］	120	60	30	5

### Exceptions and flexible approaches

If a mutagenic impurity is chemically identical to substances that are naturally present in the body (e.g., formaldehyde, acetaldehyde, hydrogen peroxide) or that occur widely in food or the environment, and the background human exposure from these sources is significantly higher than the potential pharmaceutical exposure, a higher AI may be scientifically justified. Relevant examples and guidance are provided in the addendum to ICH M7(R2) [6]. Furthermore, in cases involving serious or life-threatening diseases, late-onset chronic conditions, or limited therapeutic alternatives, an elevated AI value may be justified on a case-by-case basis, provided that the overall benefit–risk balance remains favorable and the rationale is clearly documented.

Conversely, for highly potent carcinogens belonging to the COC—such as aflatoxin-like, N-nitroso, or alkyl-azoxy compounds—the AI is expected to be far below the standard TTC level. When carcinogenicity data are available, such impurities should be controlled using AI values derived from a linear extrapolation of the tumor incidence data corresponding to an excess lifetime cancer risk of 1 in 100,000 (10^− 5^). If no carcinogenicity data exist, the AI may be estimated conservatively from structurally related analogues with similar potency, though careful expert judgment and justification are required in each case. When the impurity is a metabolite of the API, a comparison should be made between the exposure level of the metabolite formed from the API and that of the impurity. If the impurity exposure is negligible compared with the endogenous exposure to the same metabolite, the safety of the impurity can be supported by the mutagenicity data already available for the API. This is because metabolites are generally considered within the overall nonclinical safety evaluation of the parent drug.

These management principles apply to all routes of administration, and revision of AI values based on administration route is generally unnecessary unless route-specific carcinogenicity data indicate a higher risk. Given the conservative nature of the M7 approach, the AI limits are regarded as protective for all patient populations, including pediatric subjects.

## Relationship between ICH M7 and ICH Q3A/B guidelines

The ICH M7 guideline provides the most up-to-date scientific framework for evaluating and controlling potential mutagenic impurities in pharmaceuticals. It requires that any impurity with structural alerts or other evidence suggesting mutagenicity be assessed and controlled to ensure safety, regardless of its level relative to the qualification thresholds defined in ICH Q3A and Q3B [1, 2]. Therefore, even if an impurity level is below the qualification threshold specified in ICH Q3A/B, an additional safety evaluation for mutagenic potential may still be necessary under ICH M7 if structural alerts are identified. Conversely, if the impurity is demonstrated to be non-mutagenic—through Ames testing, reliable QSAR predictions, or structural analysis—then no further assessment is required.

**Fig. 2:**
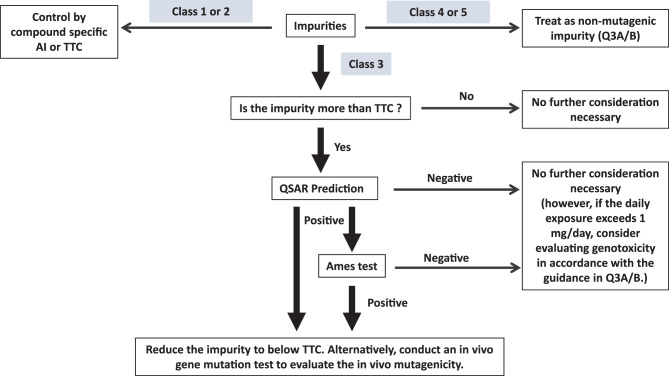
Calculation of acceptable intake (AI) and permitted daily exposure (PDE). The AI is calculated using linear extrapolation from the TD₅₀ to the human exposure level corresponding to a lifetime excess cancer risk of 10^− 5^. The PDE is calculated by dividing the non-observed effect level (NOEL) by an appropriate uncertainty factor (usually 100). The threshold value represents the same level of carcinogenicity as the untreated control

If, however, the projected daily intake of an impurity exceeds 1 mg/day during long-term administration, additional genotoxicity testing should be considered in accordance with the principles outlined in ICH Q3A/B. In such cases, chromosomal aberration tests or other complementary in vitro tests may be required to ensure that the impurity does not present additional genotoxic concern [1, 2]. On the other hand, when the daily intake of the impurity is less than 1 mg/day, and mutagenic potential has been properly assessed under ICH M7, no further genotoxicity testing is generally required. A decision tree summarizing the overall framework for assessment and control mutagenic impurities under ICH M7 is presented in Fig. 2.

## Revisions of the ICH M7 guideline

### Development of addendum (R1 and R2)

The ICH M7 guideline stipulates that, in principle, mutagenic impurities lacking carcinogenicity data should be controlled using the TTC of 1.5 μg/day. However, when reliable carcinogenicity data are available for a given mutagenic impurity, a compound-specific AI can be established using a lifetime excess cancer risk level of 10^− 5^ (1 in 100,000). In such cases, data from internationally recognized organizations, such as the World Health Organization (WHO), may be used to support AI derivation. Because the criteria and datasets used for deriving AI values could vary among pharmaceutical companies, differences in the calculated AI levels might occur, potentially leading to regulatory inconsistencies. To address this issue, the ICH M7 Expert Working Group (EWG) developed a harmonized addendum titled “Application of the Principles of the ICH M7 Guideline to Calculation of Compound-Specific Acceptable Intakes.”

The M7(R1) revision (March 2017) introduced this addendum, listing 14 compounds frequently encountered as impurities in pharmaceuticals, together with their compound-specific AI values and the corresponding derivation methods [4]. The M7(R2) revision (April 2023) further added seven new compounds and updated certain sections of the main text [5, 6]

The AI derivation process begins with a comprehensive literature review to identify the most appropriate TD₅₀ value which are typically obtained from the Carcinogenic Potency Database (CPDB) or from published studies conducted using similar methodologies [31]. AI values are then calculated using linear extrapolation from the TD₅₀ to the human exposure level corresponding to a lifetime excess cancer risk of 10^− 5^. This is operationally equivalent to dividing the TD₅₀ by 50,000, as originally proposed by the FDA and adopted in the TTC concept (Fig. 1a).

Although ICH M7 primarily addresses non-threshold mutagenic carcinogens, the addendum also provides, for practical reasons, procedures to derive acceptable exposure limits for non-mutagenic carcinogens that exhibit threshold mechanisms. For these compounds, the no-observed-effect level (NOEL) identified in rodent carcinogenicity studies is combined with an appropriate uncertainty factor (generally 100) to establish the permitted daily exposure (PDE) (Fig. 1b). Additionally, when an impurity is chemically identical to a substance that is a common food component or endogenous metabolite, and the typical exposure from such sources is far greater than that from the pharmaceutical product, a higher AI limit may be justified and documented in the addendum.

### Revisions to the main text (R2)

In the M7(R2) revision, the main body of the guideline was also updated to reflect changes in medical practice [5]. Specifically, in Table 2—which lists examples of clinical use scenarios and corresponding AI levels for different treatment durations—the example of HIV treatment was moved from the category “> 1 to 10 years” to “> 10 years to lifetime.” The associated footnotes were revised accordingly. This change reflects the fact that advances in treatment have led to treatment durations exceeding 10 years for many patients, significantly improving the long-term prognosis for people with HIV.

### Development of the questions and answers (Q&A) document

Several years after the initial implementation of ICH M7, variations in interpretation among regulatory authorities and industry stakeholders were observed. To promote consistent understanding and practical application, the M7 EWG conducted a comprehensive survey and subsequently developed a Questions and Answers (Q&A) document, which was officially adopted in May 2022 [6].

The purpose of the Q&A is to provide clarifications and detailed explanations regarding the assessment and control of DNA-reactive (mutagenic) impurities, particularly concerning information to be submitted during drug development, marketing authorization, and drug master file submissions. It aims to facilitate regulatory convergence, enhance transparency, and support global harmonization in the implementation of ICH M7. The Q&A document consists of 25 individual questions and answers [6]. This comprehensive Q&A has become an essential interpretive companion to ICH M7, providing practical guidance for regulators and industry alike and ensuring that implementation remains scientifically sound and globally consistent.

## N-Nitrosamine impurities

In July 2018, the antihypertensive drug Valsartan, manufactured by a Chinese API supplier, was found to contain N-nitrosodimethylamine (NDMA) and N-nitrosodiethylamine (NDEA)—both highly potent mutagenic carcinogens belonging to the COC [32]. This discovery triggered a global recall of Valsartan products. Subsequently, N-nitrosamine impurities were also detected in other pharmaceuticals such as Ranitidine (H₂-receptor antagonist) and Metformin (antidiabetic agent), leading to additional recalls worldwide [33]. Rodent carcinogenicity studies for NDMA and NDEA have been conducted, and the compound-specific AI values derived from their TD₅₀ data are 0.0959 μg/day for NDMA and 0.0265 μg/day for NDEA, respectively. These values are several orders of magnitude lower than the generic TTC value of 1.5 μg/day for mutagenic impurities, reflecting their extremely carcinogenic potential.

Since 2019, numerous other low-molecular-weight N-nitrosamines, such as N-nitroso-N-methyl-4-aminobutyric acid (NMBA), N-nitrosomethylphenylamine (NMPA), N-nitrosodibutylamine (NDBA), and N-nitrosodiisopropylamine (NDIPA), have been identified as pharmaceutical impurities. In addition, drug-substance-related N-nitrosamines (NDSRIs)—formed by nitrosation of the parent API (e.g., N-nitrosovarenicline, N-nitrosonortriptyline)—have been reported [34]. For low-molecular-weight N-nitrosamines such as NMBA, NMPA, NDBA, and NDIPA, AI values are typically assigned based on structural similarity (read-across) to NDMA or NDEA, resulting in the use of either 0.0959 μg/day or 0.0265 μg/day as appropriate. While a NDSRI also allows for compound-specific AI values to be assigned using read-across analysis of carcinogenicity test data for the structurally related other NDSRIs, in many cases the available information is limited. In such cases, a new method called the Carcinogenic Potency Classification Approach (CPCA) can be introduced [35, 36]. The CPCA quantitatively evaluates the carcinogenic potency of N-nitrosamines by scoring structural features that influence metabolic activation or deactivation and by integrating this with existing carcinogenicity data from known N-nitrosamines. This approach allows classification of N-nitrosamines into potency categories and enables assignment of corresponding AI values consistent with empirical carcinogenicity data. Although the CPCA remains a provisional framework, it has been formally accepted by major regulatory authorities worldwide (including EMA, FDA, and MHLW in Japan) as a scientifically robust method for estimating AI values for NDSRIs pending compound-specific carcinogenicity data [37–39].

## Concluding remarks

Following the adoption of ICH M7(R2) in April 2023 (Step 4), a new M7(R3) Maintenance Work Plan was issued in February 2024. Subsequently, during the ICH meeting held in Fukuoka in June 2024, a new topic on N-nitrosamine impurities—now recognized as a major global issue in pharmaceutical development—was formally adopted. This new initiative aims to establish a harmonized and stepwise approach for assessing the safety and appropriate control of N-nitrosamine impurities through an official ICH process, and to develop a new addendum (M7(R3)) providing a unified set of AI values based on the M7 maintenance procedure. The corresponding EWG was organized and began discussions from June 2024 [40].

It is important to recognize, however, that compounds such as NDMA and related N-nitrosamines are ubiquitous environmental carcinogens, commonly found in everyday life—for example, in grilled meat, bacon, beer, and other food items [41]. Moreover, NDMA can be endogenously formed in the human stomach through chemical reactions between dietary secondary amines (from meat) and nitrite (from vegetables). The total exposure to N-nitrosamines from such general dietary and physiological sources is estimated to exceed the AI limits established for pharmaceutical impurities [42].

In October 2024, Dr. Bruce N. Ames, the developer of the Ames test in the 1970s, passed away [43]. From the 1980s onward, Dr. Ames emphasized that the carcinogenic potential of synthetic chemicals had been overestimated, arguing that the main causes of cancer lie instead in natural dietary carcinogens, lifestyle factors, smoking, and high-fat diets, rather than trace synthetic pollutants [44]. He also contended that overregulation of synthetic chemicals might not lead to substantial improvements in public health [42]. If Dr. Ames were alive today, one might wonder how he would view the current intense regulatory debate on N-nitrosamines under ICH M7.

While stringent AI limits undoubtedly promote higher pharmaceutical quality, it remains essential from a Regulatory Science perspective to maintain a balanced and human-centered approach-one that harmonizes risk management with societal benefit and supports the ultimate goal of advancing public health. The author hopes that future revisions of ICH M7 will embody this philosophy, ensuring that the guideline continues to serve both scientific integrity and the well-being of humanity.

## Data Availability

The data supporting this review are publicly available on the ICH website

## Abbreviations

API: Active pharmaceutical ingredient
ICH: International Council for Harmonization of Pharmaceuticals for Human Use
AI: Acceptable intake
Q&A: Questions and answers
EMA: European Medicines Agency
CHMP: Committee for Medicinal Products for Human Use
FDA: Food and Drug Administration
PhRMA: Pharmaceutical Research and Manufacturers of America
QSAR: Quantitative structure–activity relationship
TTC: Threshold of toxicological concern
COC: Cohorts of concern
OECD: Organization of Economic Co-operation and Development
GLP: Good Laboratory Practice
TGR: Transgenic rodent gene mutation
ecNGS: Error-corrected next-generation sequencing
LTL: Less-than-lifetime
WHO: World Health Organization
EWG: Expert Working Group
CPDB: Carcinogenic Potency Database
NOEL: No-observed-effect level
PDE: Permitted daily exposure
NDSRIs: Drug-substance-related N-nitrosamines
CPCA: Carcinogenic Potency Categorization Approach
MHLW: Ministry of Health, Labour and Welfare
